# The Effect of Maternal Parity on Preterm Birth Risk in Women with Short Mid-Trimester Cervical Length: A Retrospective Cohort Study

**DOI:** 10.3390/jcm13164773

**Published:** 2024-08-14

**Authors:** Einav Kremer, Elyasaf Bitton, Yossef Ezra, Roie Alter, Doron Kabiri

**Affiliations:** Department of Obstetrics and Gynecology, Medical Center and Faculty of Medicine, Hebrew University of Jerusalem, P.O. Box 12000, Jerusalem 9112001, Israel

**Keywords:** premature birth, nulliparity, mid-trimester cervical length

## Abstract

**Objectives**: To evaluate the effect of maternal parity on the association between mid-trimester cervical length and preterm birth to elucidate the potential intricacies of this relationship. **Methods**: A retrospective cohort study using Electronic Medical Records (EMR) data. The study population included pregnant women with a singleton fetus and a short mid-trimester cervical length, recorded in the EMR system at a large health maintenance organization. Women were categorized by parity in the current pregnancy, and a statistical analysis was conducted to examine the relationship between parity and premature delivery. **Results**: Data were collected from 1144 records of cervical length measurements of 738 pregnancies obtained from the HMO database. The study population consisted of 259 nulliparous women (35.1%), 451 multiparous women (61.1%), and 28 grand multiparous women (3.8%). The results from the multivariate analysis of the primary outcome showed that nulliparity was significantly associated with an increased risk of premature delivery, with a risk of 1.557 for nulliparous women compared to parous women. **Conclusions**: In this study, a statistically significant association was found between nulliparity and preterm birth among women with a short mid-trimester cervical length. Nulliparous women were found to have a higher risk of preterm birth in the current pregnancy compared to parous women. Further research is needed to understand the underlying mechanisms and to develop targeted interventions to reduce the risk of premature birth in this population. These findings highlight the need to consider nulliparity as a potential risk factor in the management of pregnancies with a shortened cervix.

## 1. Introduction

Preterm birth is a major public health concern worldwide, as it remains a leading cause of neonatal mortality and morbidity [1]. Preterm birth is a syndrome driven by multiple pathological inflammatory processes. These processes involve Toll-like receptors that trigger the production of chemokines, cytokines, prostaglandins, and proteases, which collectively contribute to cervical shortening and the onset of parturition [2]. Identifying risk factors associated with preterm birth is crucial for the development of effective preventive and management strategies. Transvaginal ultrasound measurement of cervical length has been extensively investigated as a potential predictive marker for preterm birth, with evidence consistently suggesting that a shorter cervical length is associated with an increased risk [3,4]. Although several studies have investigated the association between a short mid-trimester cervix and premature birth, there remains a notable dearth of research specifically examining the influence of maternal parity on this association, including the potential role of parity order in predicting the risk of preterm birth in women with a short cervix.

The early identification of individuals at risk for preterm birth presents an opportunity to initiate interventions promptly, potentially improving maternal and neonatal outcomes.

Micronized progesterone has demonstrated efficacy in reducing the risk of preterm birth by 41% in selected high-risk populations [5,6,7,8,9,10,11]. Cervical cerclage is another preventive measure that can be utilized in women with a history of preterm birth and a short cervical length [12,13,14]. While the use of 17 alpha-hydroxyprogesterone-caproate (17OHPC) injections for -preventing preterm birth has been studied, the conclusive evidence regarding its effectiveness remains inconclusive, leading to a decline in its utilization in recent years and the withdrawal of its FDA approval [15,16,17].

Pregnant individuals can be categorized into three distinct groups based on their parity status: nulliparous (lacking any prior deliveries), multiparous (having experienced 1–5 deliveries), and grand multiparous (having undergone 6 or more deliveries). Maternal parity, denoting the count of previous deliveries encountered by a woman, stands as an additional prospective risk factor for preterm birth [18]. However, the precise interplay between maternal parity and preterm birth in individuals exhibiting a short mid-trimester cervical length remains relatively underexplored. An examination of this intricate relationship holds potential in identifying women at high risk of preterm birth based on their parity, who might benefit from targeted preventative interventions tailored to the needs of this high-risk cohort.

In this study, our objective was to assess the influence of maternal parity on the association between the mid-trimester cervical length and the occurrence of preterm birth. Should the null hypothesis be refuted, implying that parity exerts no influence on the predictive capacity of cervical length for preterm birth, a critical reevaluation of progesterone utilization in these individuals and a reassessment of the cost–benefit equilibrium associated with such treatment may be warranted. Conversely, if parity is revealed to significantly impact the association between cervical length and preterm birth, it may necessitate the implementation of more robust interventions targeting women characterized by an elevated risk profile.

Hence, the research hypothesis postulates that maternal parity plays a modulatory role in the link between cervical length and the occurrence of preterm birth. Specifically, it suggests that nulliparous women exhibiting a shortened cervix during the mid-trimester of pregnancy face a notably heightened risk of preterm birth when juxtaposed with their multiparous counterparts.

## 2. Materials and Methods

This is a retrospective cohort study using Electronic Medical Records (EMR) data to evaluate the impact of maternal parity on the intricate interplay between mid-trimester cervical length and the occurrence of preterm birth. The study population includes pregnant women aged 18 years or older with a singleton fetus and a mid-trimester cervical length measurement of 25 mm or less obtained between weeks 18 and 24, recorded in the electronic medical records (EMR) system at a large health maintenance organization (HMO) from 1 January 2012 to 31 December 2021. Notably, the focal point of analysis rests upon women who delivered after week 22 of gestation. Cervical length measurements were performed as a routine practice in mid-trimester ultrasound screening or according to a patient’s symptoms. Exclusion criteria included cases lacking recorded gestational age at delivery, instances of pregnancy termination or delivery occurring before week 22, as well as pregnancies characterized by multiple gestations.

Women were systematically classified based on the sequential order of their parity, considering both continuous and categorical variables (nulliparous, multiparous, and grand multiparous) within the present pregnancy. Cervical length assessment adhered strictly to standardized protocols and guidelines, as delineated in the International Society of Ultrasound in Obstetrics and Gynecology’s authoritative position paper [19].

Upon obtaining requisite approval from the institutional ethics committee at the HMO, a comprehensive extraction of data ensued from the electronic medical records, with due diligence exercised to ensure full anonymization, thereby upholding patient confidentiality. The compiled dataset encompassed a spectrum of pertinent demographic and obstetric background information, including maternal age, gestational week at the time of measurement, and the corresponding cervical length. Additionally, variables such as body mass index (BMI), gravidity, parity, history of previous miscarriages, records of cervical surgery, history of premature delivery, gestational age at delivery, administration of progesterone treatment, utilization of tocolysis treatment, application of antenatal corticosteroids (betamethasone), deployment of cerclage or pessary, and the gestational week of delivery within the index pregnancy were also encompassed within the dataset.

The aim of this study was to investigate the impact of parity, serving as the primary independent variable, on the occurrence of preterm birth within the subset of women characterized by a shortened cervical length. To this end, comprehensive data encompassing general, demographic, and obstetric factors concerning the study participants were systematically gathered. The acquisition of this dataset aimed to account for potential confounding variables while simultaneously identifying supplementary factors that may potentially be associated with the manifestation of preterm birth. Subsequent to data collection, a rigorous statistical analysis was conducted, affording the opportunity to predict the subset of women characterized by a shortened cervical length who exhibit an elevated susceptibility to preterm birth.

The statistical analyses were carried out utilizing IBM SPSS statistics, version 28. For the comparison of quantitative variables between two independent groups, the two-sample *t*-test was applied. For a large sample (especially above 100 cases), even if the data are not normally distributed, the probability distribution of the mean is normal, thus allowing the use of the *t*-test. The association between two categorical variables was evaluated by using either the Chi-Square test or Fisher’s exact test. The multivariate logistic regression model was applied to simultaneously assess the effect of several independent variables on the dichotomous dependent variable. This was performed using the Stepwise, Forward, Likelihood ratio approach. All statistical tests were two-tailed, and a significance level of *p* < 0.05 was deemed statistically significant.

## 3. Results

**Figure 1:**
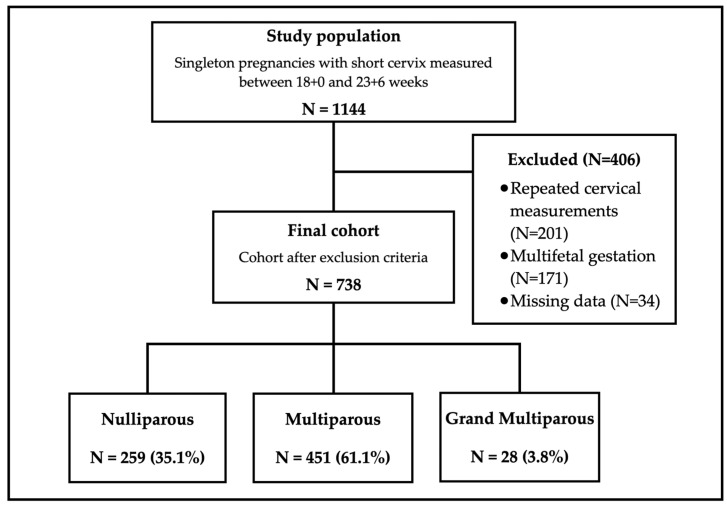
Study population and group assignment.

Figure 1 provides a comprehensive overview of the study sample characteristics and the allocation of participants into respective groups for subsequent statistical analysis. The dataset encompassed 1144 records of cervical length measurements derived from the database of a healthcare maintenance organization (HMO). These measurements were specifically restricted to the mid-trimester cervical length measured 25 mm or less. Following the exclusion of duplicate measurements originating from the same pregnancy, as well as records with missing data, the final analytical sample comprised 738 pregnancies. Within this sample, the study population comprised 259 nulliparous women (35.1%), 451 multiparous women (61.1%), and 28 grand multiparous women (3.8%), the latter being defined as individuals who had undergone six or more previous childbirths. Considering the relatively limited representation of grand multiparous individuals within the sample, it was deemed appropriate to merge this group with the multiparous cohort for subsequent analysis. Consequently, the study population was categorized into two distinct groups for analysis: nulliparous and multiparous/grand multiparous.

Table 1 presents the demographic and clinical characteristics of the study population, along with the examination of the association between each variable and the occurrence of premature delivery in the present pregnancy. Notably, no statistically significant differences were observed between the groups concerning variables such as body mass index (BMI), gravidity, parity, abortion history, cervical length measurement, or prior cervical surgery. However, significant differences were found between the groups observed in terms of age and the presence of a history of premature delivery.

Table 2 delineates the interventions administered to the study participants and their corresponding association with the outcome of premature delivery in the current pregnancy.

### 3.1. Primary Outcome: The Association of Parity with the Occurrence of Premature Delivery

An extensive statistical analysis was performed to investigate the association between parity (nulliparous, multiparous, and grand multiparous) and premature delivery. The primary outcome analysis, which compared nulliparous individuals to those classified as multiparous or grand multiparous, yielded a *p*-value of 0.066, suggesting a tendency toward an association between nulliparity and premature delivery. However, this observed difference did not reach statistical significance (Table 3).

**Figure 2:**
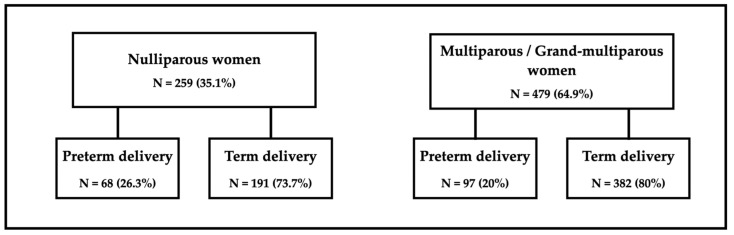
Rates of term and preterm birth by parity.

Subsequently, the groups were further divided into nulliparous, multiparous, and grand multiparous categories, and the relationship remained statistically insignificant, with a *p*-value of 0.096. Considering the limited sample size of the grand multiparous group (*n* = 28, accounting for 3.8% of the total sample), a decision was made to combine it with the multiparous group for subsequent analysis. Figure 2 graphically displays the rates of term and preterm birth among the combined group of multiparous and grand multiparous women, as well as among nulliparous women. Findings from the univariate analysis indicated that nulliparous women exhibited higher rates of preterm birth compared to multiparous/grand multiparous women. The percentages and counts of preterm deliveries were 20% (97/479) for multiparous/grand multiparous women and 26.3% (68/259) for nulliparous women. The statistical analysis included univariable comparisons and multivariable logistic regression, with the latter showing a significant association between nulliparity and preterm birth (adjusted odds ratio = 1.55, *p* < 0.001).

### 3.2. Multivariate Logistic Regression

A comprehensive multivariate analysis was employed to evaluate the influence of various independent variables on the binary outcome of preterm birth. The logistic regression model utilized a Stepwise, Forward, Likelihood ratio method. All the independent variables that were found to be significantly associated with preterm birth in the univariate analysis were included in the model as ‘candidates’ for explaining preterm birth. In the initial stage of the stepwise model, maternal age, history of premature delivery, and three specific treatments (tocolysis, pessary, and cerclage) were included as independent variables. Subsequently, delivery status (categorized as nulliparity: yes/no) was incorporated as an independent variable in the subsequent stage. Notably, even after accounting for the effects of the other independent variables, nulliparity retained its significance as a predictive factor for delivery status in the final model (Table 4).

Utilizing a multivariate logistic regression model, we identified several parameters that exhibited significant associations with an elevated risk of premature delivery among women with a shortened cervix. The findings are as follows: (1) Nulliparous women (nullipara) demonstrated a 2.02-fold increased risk of premature delivery in the current pregnancy compared to parous women. (2) A history of previous premature delivery was linked to a 2.79-fold increase in the risk of premature delivery in the current pregnancy. It is important to note that the variable of previous history of premature delivery is applicable solely to the group of women who have undergone a previous birth and is not relevant to nulliparous women. (3) Increasing age exhibited a 1.044-fold increase in the risk of premature delivery for each additional year. (4) Treatment with tocolytics demonstrated a 2.25-fold increase in the risk of premature delivery. However, caution should be exercised when determining causality, as there is a possibility of reverse causality. This implies that the need for tocolysis treatment may be a consequence of the risk of premature delivery rather than a cause. It is crucial to interpret these findings with prudence, considering the potential confounding factors and the complex nature of causal relationships in this context.

In the subsequent analysis, our focus shifted to exploring the effects of variables that exhibited statistical significance while excluding different treatments and the variable ‘history of premature delivery’ from the model. After accounting for other variables, as presented in Table 5, we identified several factors that elevate the risk of premature delivery among women with a shortened cervix: (1) Age: For each additional year of maternal age, the risk of premature delivery increases by 1.041. (2) When the reason for cervical length measurement was threatened labor, the risk of premature delivery in the current pregnancy rises by 1.480. (3) Nulliparity amplifies the risk of premature delivery in the current pregnancy by 1.557 compared to parous women.

## 4. Discussion

In this study, involving 738 women with a short cervical length during mid-trimester, a statistically significant correlation was identified between nulliparity and preterm birth. The results demonstrated that nulliparous women had a higher risk of preterm birth in the current pregnancy (adjusted odds ratio of 1.557, 95% confidence interval [CI] 1.078–2.248) compared to parous women. Additionally, two other factors demonstrated a significant association with preterm birth. Maternal age exhibited an adjusted odds ratio of 1.041 (95% CI 1.012–1.071) for each additional year, indicating that an increased maternal age augmented the risk of preterm birth. Furthermore, the presence of threatened labor was also found to be significantly associated with preterm birth, presenting an adjusted odds ratio of 1.480 (95% CI 1.038–2.111). These findings emphasize the importance of considering nulliparity, maternal age, and threatened labor as crucial factors in assessing the risk of preterm birth in women with a short mid-trimester cervical length.

Numerous studies have explored the association between a short mid-trimester cervix and premature birth; however, there is a paucity of research specifically examining the impact of parity and the predictive value of parity order (nulliparous, multiparous, and grand multiparous) in relation to this association.

The current study revealed that a previous occurrence of premature birth serves as a noteworthy risk factor for premature birth in the current pregnancy. This finding aligns with prior well-conducted research, which consistently indicates that a history of premature birth poses a risk for subsequent pregnancies.

### Strength and Limitations

This study exhibits several noteworthy strengths, including a substantial cohort of 1144 cervical length examinations, with a final study population comprising 738 women. The study’s findings demonstrate statistical significance and exhibit robustness, thereby enhancing the validity of the conclusions drawn.

Nevertheless, it is essential to acknowledge the presence of certain limitations in this study. Firstly, the retrospective design entails reliance on data extracted from medical records. Since these records were generated by multiple healthcare providers, variations in the reporting process and the potential for bias may exist. Furthermore, some records contained missing data, necessitating the inclusion of information obtained from hospitalizations and primary care visits. Given the retrospective nature of the study and the inconsistent and non-uniform documentation in the records, there is a possibility of incomplete data within the dataset. Additionally, the data did not allow for differentiation between spontaneous and medically indicated preterm births, nor did they consistently provide comprehensive neonatal characteristics. It is crucial to recognize the potential influence of various treatments administered to individuals with a short cervix, such as progesterone, tocolytics, steroids, cervical cerclage, and pessary, on the gestational age at delivery and the primary outcome measure. These factors possess the potential to impact the study’s results and must be considered when interpreting the findings.

This study has revealed a significant association between nulliparity and an elevated risk of premature birth in the presence of a shortened cervix. However, the precise mechanisms underlying this relationship are not fully understood. Therefore, further research is needed to investigate potential causal factors and clarify this association. There are several potential explanations for this relationship. One possibility is that nulliparous women may exhibit structural or functional differences in their reproductive tract that heighten their susceptibility to premature birth. These differences may be related to the cervix, uterus, or other reproductive structures. Additionally, hormonal or environmental factors might contribute to the increased risk of premature birth in nulliparous women with a shortened cervix [2]. Immunological factors could also play a role, as nulliparous women may exhibit a more aggressive or less well-regulated immune response, potentially triggering premature labor. It is important to note that these explanations are merely a few among several possibilities, and comprehensive research is required to fully comprehend the underlying mechanisms involved.

The findings of this study carry significant clinical implications. The increased adjusted odds ratio of preterm delivery observed in nulliparous women emphasizes the importance of considering nulliparity as a potential risk factor in the management of pregnancies involving a shortened cervix. Future research should prioritize the identification of specific mechanisms that underlie this association and the development of targeted interventions aimed at mitigating the risk of premature birth in nulliparous women with a shortened cervix. Such efforts have the potential to enhance clinical decision making and improve outcomes for this specific population.

## 5. Conclusions

This study established a significant link between nulliparity and an elevated risk of premature birth in women with a shortened cervix. After accounting for potential confounding variables, nulliparous women demonstrated a 1.557-fold higher risk of premature birth compared to parous women. The validity of this association was further reinforced by statistically significant associations with age, history of preterm birth, and threatened labor. These findings underscore the importance of recognizing nulliparity as a potential risk factor for premature birth and emphasize the necessity for additional research to unravel the underlying mechanisms involved. Furthermore, there is a critical need to explore potential interventions that can effectively mitigate the risk of premature birth in this specific population.

## Figures and Tables

**Table 1:** Demographic characteristics of the study population by parity.

Variable	Term Birth *n* = 573	Preterm Birth *n* = 165	Study Population *n* = 738	*p*-Value
Maternal age	30.18 ± 6.17	31.66 ± 6.58	30.51 ± 6.29	0.008
BMI	27.055 ± 5.38	26.594 ± 5.65	26.952 ± 5.44	0.341
Total Pregnancies	3.23 ± 2.39	3.09 ± 2.36	3.2 ± 2.38	0.498
Total Miscarriages	0.81 ± 1.27	0.77 ± 1.16	0.8 ± 1.25	0.729
Cervical length mean	16.714 ± 7.12	16.985 ± 6.28	16.775 ± 6.93	0.659
History of D&C	139 (23.1%)	26 (19.3%)	165 (22.4%)	0.339
History of cervical surgery	153 (21.7%)	12 (35.3%)	165 (22.4%)	0.064
History of preterm deliveries (among multiparity and grand multiparity)	57 (15.9%)	40 (33.3%)	97 (20.3%)	*p* < 0.001

**Table 2:** Interventions and their association with premature delivery.

Interventions	Term Birth *n* = 573	Preterm Birth *n* = 165	*p*-Value
Progesterone	374 (65.3%)	115 (69.7%)	0.289
Tocolytic agents	46 (8%)	27 (16%)	0.002
Steroids for lung maturation	109 (19%)	43 (26%)	0.049
Cerclage	61 (10%)	31 (18.8%)	0.005
Pessary	14 (2.4%)	10 (6%)	0.021

**Table 3:** Parity and risk of premature delivery.

Characteristics	Term Delivery *n* = 573	Preterm Delivery *n* = 165	*p*-Value
Parity mean SD	1.42 ± 1.68	1.30 ± 1.84	0.066

**Table 4:** Multivariate logistic regression of factors associated with preterm birth.

Parameters	*p*-Value	Adjusted OR (CI of OR)
Age	0.003	1.044 (1.015–1.075)
History of preterm delivery	*p* < 0.001	2.788 (1.718–4.526)
Tocolytic agents treatments	0.003	2.253 (1.325–3.830)
Cerclage	0.060	1.611 (0.980–2.650)
Pessary	0.075	2.184 (0.924–5.160)
Nulliparity	0.001	2.022 (1.336–3.061)

**Table 5:** Risk factors for premature delivery in women with shortened cervix: multivariate limited logistic analysis results.

Parameters	*p*-Value	Adjusted OR (CI of OR)
Age	0.005	1.041 (1.012–1.071)
Cervical measurement reason	0.189	1.647 (0.782–3.470)
History of cervical surgeries	0.030	1.480 (1.038–2.111)
Nulliparity	0.018	1.557 (1.078–2.248)

## Data Availability

Data will be available upon request from interested researchers.
